# Common Fragile Sites: Genomic Hotspots of DNA Damage and Carcinogenesis

**DOI:** 10.3390/ijms130911974

**Published:** 2012-09-20

**Authors:** Ke Ma, Li Qiu, Kristin Mrasek, Jun Zhang, Thomas Liehr, Luciana Gonçalves Quintana, Zheng Li

**Affiliations:** 1 Department of Biochemistry and Molecular Biology, Tianjin Medical University Cancer Institute and Hospital, Tianjin 300060, China; E-Mails: nmgdxmark@126.com (K.M.); nangongningchen@163.com (L.Q.); doctorjunzhang@163.com (J.Z.); 2 Key Laboratory of Ministry of Education for Breast Cancer Prevention and Treatment, Tianjin Medical University Cancer Institute and Hospital, Tianjin 300060, China; 3 Institute of Human Genetics, Jena University Hospital, Friedrich Schiller University, D-07743 Jena, Germany; E-Mails: kmra@mti.uni-jena.de (K.M.); thomas.liehr@mti.uni-jena.de (T.L.); luciana.quintana@mti.uni-jena.de (L.G.Q.)

**Keywords:** replication, instability, CFS, cancer, *FATS*, checkpoint

## Abstract

Genomic instability, a hallmark of cancer, occurs preferentially at specific genomic regions known as common fragile sites (CFSs). CFSs are evolutionarily conserved and late replicating regions with AT-rich sequences, and CFS instability is correlated with cancer. In the last decade, much progress has been made toward understanding the mechanisms of chromosomal instability at CFSs. However, despite tremendous efforts, identifying a cancer-associated CFS gene (CACG) remains a challenge and little is known about the function of CACGs at most CFS loci. Recent studies of *FATS* (for Fragile-site Associated Tumor Suppressor), a new CACG at FRA10F, reveal an active role of this CACG in regulating DNA damage checkpoints and suppressing tumorigenesis. The identification of *FATS* may inspire more discoveries of other uncharacterized CACGs. Further elucidation of the biological functions and clinical significance of CACGs may be exploited for cancer biomarkers and therapeutic benefits.

## 1. Introduction

Common fragile sites (CFSs) are specific chromosomal regions that preferentially form gaps or breaks on metaphase chromosomes under replication stress. The most typical inducer of CFSs is aphidicolin, an inhibitor of DNA polymerase that induces 77 of 88 known CFSs [1–3]. Unlike rare fragile sites, which involve expansion of a repeat motif (such as CGG), and are inherited in a Mendelian manner without a direct role in cancer, CFSs are seen in all individuals and frequently correlate with cancer [1,4,5]. Intriguingly, not all CFSs form breaks or gaps at the same frequency [6], and two sites (FRA3B and FRA16D) in the human genome are most prone to form lesions. CFSs are evolutionarily conserved and late replicating regions with AT-rich sequences [7]. Genomic instability, a hallmark of cancer, occurs preferentially at CFSs. Thus, the study of CFSs can not only provide insight into carcinogenesis, but also lead to the discovery of new cancer-related genes.

Here, we will review recent advances in the field of cancer-related CFSs. We also describe the efforts in mapping FRA10F and identification of a new CFS gene, named *FATS* (for Fragile-site Associated Tumor Suppressor), which is involved in DNA damage response.

## 2. Mechanisms of CFS Instability

CFSs are part of the normal chromosomal structure, and they are normally stable in cultured cells. After partial inhibition of replication by aphidicolin, BrdU, or 5-azacytidine, CFSs are expressed at specific loci of metaphase chromosomes. Fragile sites are conserved among mammals and are also found in lower eukaryotes including yeast and fly [8]. In addition to displaying gaps and breaks, CFSs are preferably involved in sister chromatid exchange (SCE), deletions and translocations [9–12]. CFSs are also “hotspots” for gene amplification [13–15] and viral integration [16–19]. As genetic instability is a hallmark of cancer, the strong correlation between most CFSs and imbalanced loci/breakpoints in tumor genome has attracted extensive studies on the molecular basis of CFSs.

One of the features of CFS is late-replication. Sequences at FRA3B replicate very late and the treatment of aphidicolin results in a further delay in replication. Remarkably, more than 10% of FRA3B sites remain unreplicated in G_2_ phase after aphidicolin treatment [20,21]. Replication timing studies of FRA16D, FRA7H, FRA1H and FRA2G also indicate the late-replicating feature of these CFSs [22–24], which confers sensitivity to further delay in response to replication inhibitors. Palumbo *et al*. [25] analyzed the replication dynamics at FRA6E, a mid-late-replicating sequence, and observed that chromosome breakages occur preferentially at an early/late replication transition zone. Investigations of cloned CFSs reveal no sequence similarities or consensus motifs. Instead, all CFSs examined to date are comprised of AT-rich flexibility islands with the potential of forming stable secondary structures. Zlotorynski *et al.* [26] have shown that the flexible sequences at FRA7E are composed of interrupted runs of AT-dinucleotides and these sequences show similarity to the AT-rich minisatellite repeats that underlie the fragility of some rare fragile sites. Such sequences at CFS have the potential to form secondary structures to affect replication. Similarly, several regions with a potential unusual DNA structure, including high-flexibility, low-stability and non-B-DNA-forming sequences, were identified at FRA7H and FRA3B [20,27]. Thus, CFS regions contain clusters of flexibility peaks that are extremely AT-rich. Recent conformational studies of cytosine-rich CFS regions indicate that homo-C tracts in duplex DNA may be associated with DNA-protein interactions *in vivo*, predisposing certain genomic regions to chromosomal fragility [28].

A genetic assay showed that a short AT-rich region within FRA16D causes increased chromosome breakage by forming strong secondary structures that stall replication fork progression [29], supporting the argument that repeat instability is an important and unique form of mutation that is not only linked to neurodegenerative disorders caused by expansion of trinucleotide repeats at rare fragile sites [30], but also linked to CFS fragility caused by tandem repeat sequences, including AT-dinucleotide repeats. Repeat instability involved the formation of unusual DNA structures during DNA replication, repair and recombination. Based on experimental studies in the prokaryotic model system [31,32], deletions or amplifications of repeat sequences tend to occur during DNA replication in a leading/lagging strand-dependent manner. Stably transfected FRA3B sequences in HCT116 cells exhibit instability at ectopic sites [33]. More recently, Letessier *et al*. [34] showed that FRA3B instability in lymphocytes relies on a paucity of initiation events rather than on fork slowing or stalling, thus, confirming impaired replication dynamics at FRA3B [35]. Helmrich *et al*. [36] reported that long genes, such as *FHIT*, *WWOX* and *IMMP2L*, exhibit CFS instability only when they are transcribed. Also, regions of concomitant transcription and replication in late S phase exhibit CFS, and RNA:DNA hybrids (R-loops) form at sites of transcription/replication collisions independently of aphidicolin treatment. This report not only highlights that CFSs are hotspots of DNA damage but also suggests that functional replication machinery must be involved in the resolution of conflicts of transcription and replication machineries to ensure genomic stability [36]. Moreover, Kerem’s laboratory found that even under normal growth conditions, replication fork progression at FRA16C is slow and forks frequently stall at AT-rich sequences. Unlike in the entire genome, additional origins in FRA16C region are not activated under mild replication stress, leading ultimately to the failure of normal replication completion in the FRA16C region [37]. It is now increasingly clear that failure of origin activation is a common feature of CFSs [38,39]. Therefore, CFS fragility is caused either by perturbed fork progression at AT-dinucleotide repeats that form stable secondary structures, or by an intrinsic paucity of replicating origins along a CFS.

Interestingly, CFSs, having been mapped most often in lymphocytes, were shown to be expressed in a cell-specific or tissue-specific manner [34,40]. This points towards the necessity that CFS contribution to tumor-specific chromosomal rearrangements need to be reassessed [34], as different chromosomal regions can be involved in fragility in different cell types; this reveals a yet disregarded potential epigenetic nature of CFSs [41].

## 3. Cancer-Associated CFS Genes

Genetic damage is considered as a hallmark of most cancer cells, and the induction of genomic instability is a crucial event in carcinogenesis. Numerous studies have shown that CFSs are genomic loci of frequent deletion, amplification and rearrangement in cancer cells. Yunis *et al.* [42] reported that fragile sites are targets of diverse mutagens and carcinogens, and 67% of the *in vitro* induced fragile sites are located in cancer-specific breakpoints. For nearly thirty years, this correspondence between the locations of fragile sites and cancer-associated loci has attracted intensive investigation aimed at identifying fragile-site-associated tumor suppressor genes or oncogenes. Most CFSs have been determined by FISH mapping. However, only a few of CFSs have been molecularly mapped to date (Table 1), largely due to the difficulties in identifying cancer associated CFS genes (CACGs) by means of positional cloning. Although numerous genes may be associated with a CFS, usually less than two genes are found as CACGs.

Most studies of CFS associated genes have focused on FRA3B and FRA16D because they are the two most frequently expressed and best-characterized fragile sites. Two tumor suppressor genes, *FHIT* and *WWOX*, are associated with FRA3B and FRA16D, respectively. FRA3B frequently exhibits allelic loss or homozygous deletions in many tumor types including lung, kidney, breast, digestive tract and lymphomas [43–47]. FRA3B is relatively small in size, only 200–300 kb at 3p14.2. In contrast, *FHIT* locus is composed of ten exons distributed over at least 500 kb. Despite its large size, *FHIT* encodes only a small 1.1 kb transcript. Aberrant transcripts of the *FHIT* locus were found in approximately 50% of esophageal, stomach and colon carcinomas [46], and loss of heterozygosity (LOH) at *FHIT* was found in 85% of primary effusion lymphoma [47], and 84% of gastric cancer [48]. FHIT is a diadenosine triphosphate (Ap3A) hydrolase [49], and its role as a tumor suppressor has been confirmed. FHIT-deficient mice have increased susceptibility to NMBA-induced tumorigenesis [50]. Interestingly, although FHIT overexpression suppresses tumorigenicity both *in vitro* and *in vivo*, the hydrolase “dead” *FHIT* mutant also suppresses tumorigenicity *in vivo* [51], indicating that the hydrolase activity of FHIT is not required for tumor suppression. Investigating the protein interactions with FHIT shed light on the mechanisms underlying FHIT-mediated suppression of tumorigenesis. Weiske *et al*. [52] showed that FHIT interacts with the *C*-terminus of β-catenin and negatively regulates transcription of target genes such as *cyclin D1*, *MMP-14* and *survivin*. In addition, ectopic activation of *FHIT* in FHIT-deficient H1299 cells significantly reduced the invasive potential of tumor cells by down-regulating expression of RhoC, a potential marker of tumor cell invasion and metastases [53].

FRA16D lies within the large *WWOX* gene, which encodes a small 1.2 kb transcript but extends over 1 Mb. The highest normal expression of *WWOX* was observed in hormonally regulated tissues such as testis, ovary, and prostate [54]. Surprisingly, although FRA16D is a LOH site in breast, lung, esophageal, gastric, pancreatic carcinomas and lymphomas [47,54–59], WWOX mRNA is overexpressed in multiple breast cancer cell lines, including MCF-7, MDA-MB-453, SKBR3 and ZR-75-1 [54]. Watanabe *et al*. [60] reported that the level of WWOX protein is elevated rather than decreased in gastric and breast carcinomas, challenging the notion that WWOX is a classic tumor suppressor. The function of WWOX as a tumor suppressor was later supported by *in vivo* evidence that inactivation of *WWOX* gene accelerates tumor progression in mice and that *WWOX* is haploinsufficient for the initiation of tumor development [61,62]. As *WWOX* gene encodes four transcript variants and current studies support the role of *WWOX* variant 1 as a tumor suppressor [63], the controversies regarding the expression of *WWOX* in cancers are probably caused by variant 4, whose function remains to be elucidated. WWOX shuttles between cytoplasm and nuclei, and cytoplasmic WWOX was associated mainly with mitochondria [60]. However, the Golgi localization of WWOX has been reported [64,65]. Ludes-Meyers *et al*. reported that WWOX normally resides in the Golgi and that aberrantly-spliced mRNAs encode WWOX protein isoforms displaying abnormal intracellular localization to the nucleus, possibly functioning as dominant negative inhibitors of full length WWOX [65]. It seems that inducing apoptosis plays a major role in WWOX-mediated suppression of tumorigenesis. WWOX induces apoptosis synergistically with p53, and WWOX phosphorylation at Tyr33 is required for WWOX binding to p53 and the pro-apoptotic function of WWOX [66]. WWOX also interacts with c-Jun proto-oncogene and suppresses c-Jun transcriptional activity [67].

Besides FRA3B and FRA16D, to date, 17 CFSs have been molecularly cloned with reports of associated genes (Table 1): FRA2G [68], FRA2H [69], FRA4F [70], FRA6E [71], FRA6F [72], FRA7B [73], FRA7G [14,74–76], FRA7I [77], FRA7K [78], FRA8C [79,80], FRA9E [81], FRA10F [82], FRA10G [83,84], FRAXB [85] and FRAXC [86,87]. Although FRA7E and FRA7H have been cloned, there are no functional reports about the associated genes. The majority of fragile sites appear to be located either at the junction of Giemsa-negative and Giemsa-positive bands or in Giemsa-negative bands close to the junction [5]. However, few CFSs have been experimentally mapped in the post-genomics era. Usually, there are multiple genes associated with a CFS. Most of early studies focused on the cloning of a CFS, trying to identify a CACG involved in carcinogenesis or cancer development. Although identifying CFS-associated genes may be easier in the post-genomics era, validating a CACG has remained challenging until now. Several CACGs have been identified, besides *FHIT* and *WWOX. PAPPA* (at FRA9E) and *PARK2* (at FRA6E) are candidate tumor suppressor genes with evidence of LOH or expression loss in cancer [71,81]. The chromosomal breakage at FRA7G is associated with amplification, deletion, and/or translocation in certain forms of cancer. The MET oncogene, a receptor kinase, is a CACG at FRA7G. The amplification of *MET* has been found in human gastric carcinoma and esophageal adenocarcinoma [14,76]. The PI3K-Akt mediates oncogenic MET-induced centrosome amplification and chromosome instability [88]. The activation of MET tyrosine kinase stimulates the survival, proliferation, and invasion of glioblastomas. Joo *et al*. [89] further showed that a distinct fraction of cells expressing a high level of MET in human primary glioblastomas multiforme (GBM) specimens are highly clonogenic, tumorigenic, and resistant to radiation. Inhibition of MET signaling in glioblastoma stem cells (GSC) disrupts tumor growth and invasiveness both *in vitro* and *in vivo*, suggesting that MET activation is linked to cancer stem cell phenotype. Interestingly, FRA7G is also a LOH site in breast, ovarian, and prostate cancer [74,75]. Tatarelli *et al*. [90] observed lack of *TESTIN* expression in 22% of cancer cell lines and 44% of the cell lines derived from hematological malignancies, suggesting that *TESTIN* may represent a candidate tumor suppressor gene at 7q31. Adenoviral transduction of *TESTIN* gene into T47D breast cancer cells promotes apoptosis and suppresses the tumorigenic potential of T47D cells in nude mice. However, *TESTIN* overexpression in MCF-7 breast cancer cells does not show pro-apoptotic effects and antitumor activity *in vivo* [91]. In addition, Han *et al*. [92] argued that *TESTIN* are co-amplified with the *MET* oncogene and overexpressed in human gastric cancer cell line GTL-16, challenging the role of *TESTIN* as a tumor suppressor gene at FRA7G. Wang’s laboratory reported that DNA breaks at FRA10G generate oncogenic *RET*/*PTC* rearrangements, which are frequently found in papillary thyroid carcinoma (PTC), in human thyroid cells [83,84]. FRA8C is a preferred site of integration for human papillomavirus (HPV) in cervical tumors, and *MYC* oncogene, which is at the boundary of FRA8C, is frequently deregulated—usually by translocation or amplification—in various tumor types [5,79,80]. It has been well established that deregulation of *MYC*-mediated signal transduction plays an important role in tumorigenesis. The major advances in our understanding of *MYC* functions have been summarized in an excellent review [93]. Differential display (DD)-PCR analysis comparing normal ovarian epithelial cultures and ovarian cancer cell lines identified pregnancy-associated plasma protein-A (*PAPPA*) as a CACG with frequent loss of expression in ovarian cancer cell lines. Fluorescence *in situ* hybridization (FISH) analysis determined that *PAPPA* is localized within the distal end of FRA9E, which is one of the largest CFS extending over 9 Mb [81]. Lack of functional *PAPPA* compromises mouse ovarian steroidogenesis and female fertility [94]. However, the functional role of PAPPA deficiency in ovarian cancer remains obscure. The *RET* proto-oncogene encodes a receptor tyrosine kinase that is required for the development of the urogenital system and the nervous systems. Recently, Gandhi *et al*. [83,84] found that *RET* fusion gene is involved in papillary thyroid carcinoma (PTC), which is a CACG at FRA10G. *RET*/*PTC* rearrangements are found in 30% to 40% of adult and 50% to 60% of pediatric *PTC* tumors, and the two most common subtypes are *RET*/*PTC1* and *RET*/*PTC3*, where *RET* is translocated with *CCDC6* and *NCOA4*, respectively [95–97]. Unexpectedly, *RET* is methylated in 27% of colon adenomas and in 63% of colorectal cancers. The aberrant methylation of *RET* correlates with decreased RET expression, whereas the restoration of RET in colorectal cancer cell lines results in apoptosis [98]. These results indicate that *RET* is a potential tumor suppressor gene in colon cancer. Interestingly, *RET* mutations have been found in both multiple endocrine neoplasia type 2, characterized by medullary thyroid carcinoma (MTC), and primary colorectal cancer [98,99], supporting its oncogenic function in thyroid carcinoma and tumor-suppressing function in colon cancer, respectively. Further understanding of the functions and physiological role of *RET* is essential to define the molecular mechanisms underlying *RET*-associated tumorigenesis. *PARK2* lies within FRA6E, a large common fragile site. *PARK2* deletions occur frequently in sporadic colorectal cancer and accelerate adenoma development in Apc mutant mice [100]. Somatic mutations of *PARK2* in glioblastoma and other human malignancies have been reported [101]. *DMD* is embedded in a CFS FRAXC and exhibits reduced expression in brain tumors [86]. Array CGH analysis and nucleotide-sequence analysis reveal multiple independent rearrangements frequently occurring in both *PARK2* and *DMD* in germ cell and cancer cell lines [87]. Notably, CFS expression varies extensively among individuals, and only two sites (FRA3B and FRA16D) were fragile in all of the tested individuals [6]. The expression frequency of some CFSs (e.g., FRA10F) is as low as 5% of the population [6]. Although CFSs are regions of genomic instability that have also been associated with deletions, translocations, and gene amplification during cancer development, recent advances in our understanding of the molecular nature of CFSs indicate that protein-coding CFS genes may not exist in all cancer-related CFSs. Brueckner *et al*. [69] fine-mapped the location of FRA2H using six-color FISH analysis and showed that it is one of the most active CFSs in the human genome. Surprisingly, FRA2H encompasses approximately 530 kb of a gene-poor region containing a novel large non-coding RNA gene. Using custom-designed array comparative genomic hybridization (CGH), gross and submicroscopic chromosomal rearrangements involving FRA2H in a panel of 54 neuroblastoma, colon and breast cancer cell lines were detected [69]. The function of this non-coding RNA gene remains to be elucidated.

**Figure 1:**
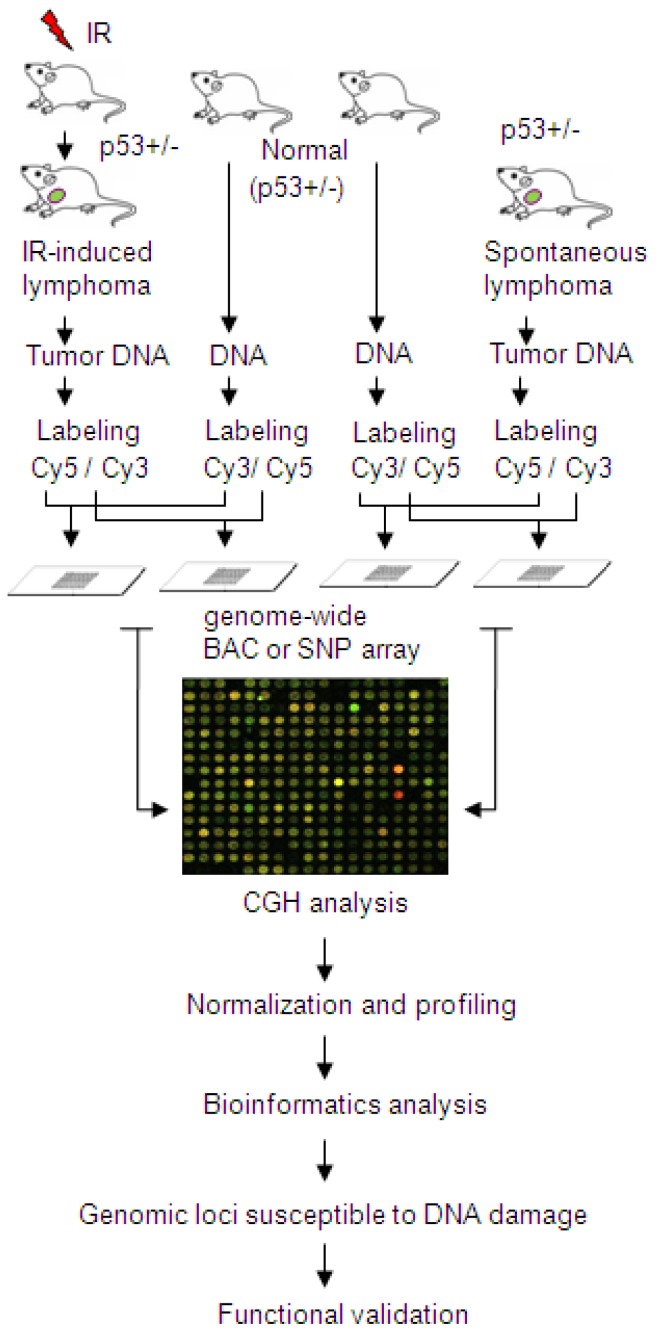
Experimental design for identification of genomic regions susceptible to DNA damage. This approach may be useful to discover evolutionarily conserved CFS genes. IR, ionizing radiation; CGH, comparative genomic hybridization; BAC, bacterial artificial chromosome; SNP, single nucleotide polymorphism.

**Figure 2:**
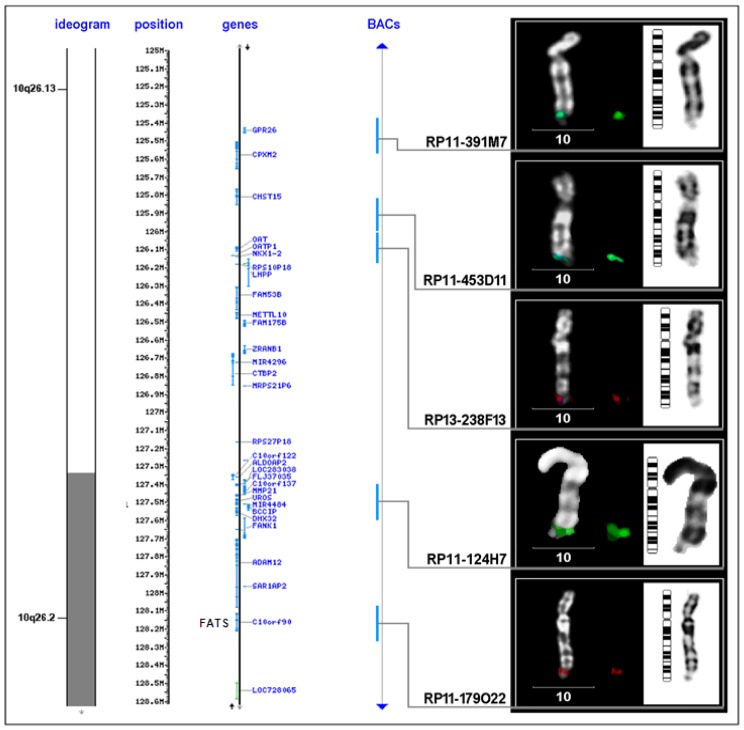
Mapping of FRA10F by FISH. The indicated BAC DNA was labeled and subjected to FISH analysis. The associated genes including *FATS* (*C10orf90*) are shown. Lymphocyte cultures were established from 1 mL of peripheral whole blood and 9 mL of RPMI 1640 medium (GIBCO) supplemented with 10% fetal bovine serum. After incubation at 37 °C for 48 h, aphidicolin solution was added (final concentration: 0.2 μM). Cell harvest and metaphase preparations followed routine cytogenetic techniques, and chromosomes were identified by DAPI (Sigma) staining. For each individual, chromosomal breakages/gaps were scored from 500 metaphases, captured and marked by coordinates using a metaphase finder. Images were obtained using a Zeiss Axioplan2 fluorescence microscope equipped with an IMAC CCD camera. FISH, fluorescence *in situ* hybridization.

**Figure 3:**
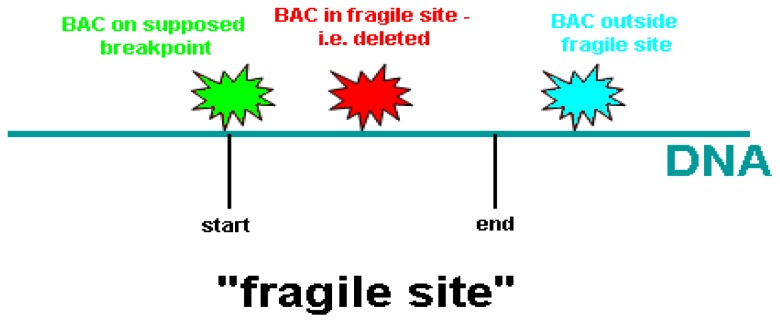
Different situations of FISH signals at a fragile site. The boundary of a fragile site was defined by FISH analysis probed with a series of labeled BAC clones. A fragile site consists of colocalized BAC DNA regions on supposed breakpoint and inside fragile site.

CFSs are specific genomic loci susceptible to DNA damage induced by replication stress and genotoxic agents. Consistently, CFSs are preferentially involved in sister chromatid exchange [9], an indication that some type of repair has occurred at the site. Ionizing radiation (IR) is a well-known carcinogen that is able to induce DNA damage and initiate neoplastic progression. In addition, IR-induced tumors exhibit a high frequency of localized deletion/amplification events [102]. It is therefore practical to identify evolutionarily conserved CFS genes through genome-wide dissecting allelic imbalances in IR-induced tumors. To this end, Li *et al*. [82] analyzed CGH profiling of IR-induced mouse lymphomas in comparison with those of spontaneous tumors (Figure 1), using the same approach as described before [102]. All the tumors had the same genetic background (p53+/−), which facilitates time-consuming collection of tumor samples [103] and high quality of CGH analysis with less interference of repetitive sequences in mouse than that in human. Using microarrays containing over 19,200 bacterial artificial chromosome (BAC) clones with insert size in the range of 170–210 kb and maximized coverage of mouse genome [104], the results of CGH analysis reveal a deletion site close to the end of mouse chromosome 7 in IR-induced tumors. This DNA region, corresponding to the contig of RP23-35I7, was deleted in 15% of spontaneous tumors (*n* = 20). Remarkably, the same region was deleted in 21 out of 29 (72.4%, *p* < 0.001) IR-induced tumor samples, indicating that this genomic region, which likely harbors an uncharacterized candidate tumor suppressor gene D7Ertd443e (Genbank accession number: GQ499374, NM_001199941), is highly susceptible to DNA damage [82]. Its human counterpart, C10orf90, a functionally uncharacterized gene, is mapped to a CFS FRA10F at 10q26, spanning a LOH region associated with cancers [105,106]. The co-localization of this new gene with FRA10F was confirmed by fluorescence *in situ* hybridization (FISH), using a BAC probe RP11-179O22 that spans 170 kb and contains all exons of *C10orf90* (Table 2). Therefore, this gene, encoding an uncharacterized and evolutionarily conserved protein, is named *FATS* (for Fragile-site Associated Tumor Suppressor) [82]. Non-coding sequences of *FATS* gene are AT-rich. Interestingly, the largest exon of mouse *FATS*, encoding the NH2-terminal domain of *FATS* that is evolutionarily conserved, is surrounded by AT-rich sequences inserted with AT-dinucleotide repeats, which is a feature of CFSs instability. Unusually, additional dinucleotide repeats such as (CA)*_n_* and (TG)*_n_*, are distributed in AT-rich sequences both upstream and downstream of this coding exon, which may form a succession of stem-loop structures with a tendency to induce replication pausing and cause DNA fragility [82]. These dinucleotide repeats inserted in AT-rich sequences thus confer genetic instability to the *FATS* locus. Moreover, FATS expression is deficient or silent not only in mouse lymphomas and multiple human cancer cell lines [82], but also in clinical tumor samples from patients with ovarian, breast and lung cancer [107–109], demonstrating that *FATS* is a CFS gene at FRA10F. Fine-mapping of FRA10F by FISH using five BAC probes defines the boundary of FRA10F (Table 2), which spans 2.8 Mb at the junction of 10q26.13–26.2 (Figure 2 and 3). Although 30 genes are associated with FRA10F, only *FATS* is functionally validated as a CACG. The approach to *FATS* identification is practical for discovering some, if not all, other uncharacterized CACGs, especially when a genome-wide high-density SNP-oligonucleotide array is commercially available. Although IR does not cause gaps and breaks at CFSs in culture cells, IR-induced DNA damage, which is associated with deletion or amplification, occurs at relatively specific genomic loci in the mouse tumor genome. The *FATS* locus is just one of the most repetitively detected loci susceptible to DNA damage [82,102]. Although there are 30 genes associated with FRA10F spanning 2.8 Mb (Figure 2), only the *FATS* gene locus is highly susceptible to DNA damage-induced deletion in p53+/− tumors [82,102]. Similarly, aphidicolin-induced replication stress leads to DNA damage, and the number of CFS induced by aphidicolin can be as many as 230, dependent on the dose of aphidicolin treatment [110]. Notably, chromosomal lesions induced by chemical mutagens involve large regions of chromosomes, and changes in spontaneous tumors often involve whole chromosomes [102]. In contrast, IR-induced tumors exhibit a high frequency of localized deletion/amplification events [102], facilitating the identification of cancer-related genes using the approach illustrated in Figure 1. Because of very high numbers of recurrent gains and losses induced by IR, the cancer-related genes identified by this approach may consist of CACGs and other oncogenes or tumor suppressor genes. In the post-genomics era, identifying CFS-associated genes and cloning CFSs is less important than identifying new CACGs with important physiological functions and a causal role in tumorigenesis. Given that CFS instability, at least FRA3B instability, exhibits tissue specificity [28], cancer-related CFSs and *CACG*s need to be reassessed in the cell type from which each tumor originates. The frequent deletion of T-cell-receptor-α (TCR-α) gene locus is both detected in both spontaneous and IR-induced tumors [102], using the approach we propose in Figure 1. In addition, microarray-based studies of mouse tumor models (Figure 1) also reveals the tumor suppressor gene *CDKN2A* (p16) at a frequently deleted region in tumors induced by IR (data not shown). These results demonstrated the reliability of the microarray-based approach in mouse tumor models, which led to the discovery of the *FATS* tumor suppressor gene. Recently, Bignell *et al*. [111] identified 2428 somatic homozygous deletions (HDs) in 746 cancer cell lines. Interestingly, *CDKN2A* (p16) exhibits more HDs than other recessive cancer genes, and TCR-α genes are located at the deepest HD in lymphoid cells in their dataset [111]. The microarray-based screening of murine tumor models (Figure 1) therefore may be helpful to discover new cancer-related genes, including some evolutionarily conserved CACGs whose gene loci are susceptible to DNA damage. After functional validation, the localization of a CACG at a CFS should be verified by FISH analysis using BAC clones spanning the whole gene locus. Similarly, Tsantoulis *et al*. [112] performed a genome-wide array CGH study on preneoplastic mouse models, in addition to analyzing 56 aphidicolin-type CFSs in growth-factor-induced human skin hyperplasia, showing that genomic alterations are more common within CFSs in epidermal and urothelial preneoplastic lesions as well as in cancer. Further functional study based on these microarray data may provide new insights into the molecular nature of uncharacterized CFSs, leading to identification of new CACGs.

## 4. CFS Expression and Defects in Checkpoint Proteins

Progression of cell cycle is an orchestrated process, and cells have evolved checkpoint mechanisms to delay cell division in response to DNA damage to allow for DNA repair, while maintaining genomic integrity by protecting dividing cells from potentially fatal consequences of DNA damage [113]. ATM and ATR kinases are important DNA damage checkpoint proteins functioning in overlapping pathways. ATM responds primarily to DNA double-strand breaks (DSBs), while ATR plays a major role in responding to stalled and collapsed replication forks. Caffeine, an inhibitor of phosphoinositide 3-kinase related kinases, including ATM and ATR, significantly increases CFS breakage in conjunction with FdU and aphidicolin [5,114]. Several mutagens and carcinogens, including benzene, carbon tetrachloride, and dimethyl sulfate, can induce fragile site breakage [42]. BrdU and 5-azacytidine also induce CFS expression at distinct loci, which are different from aphidicolin-induced CFSs [1]. Consistent with the inhibitory effect of aphidicolin on DNA polymerase, reduced levels of DNA polymerase delta induced CFS instability in yeast [115]. Short hairpin RNA-mediated depletion of Polymerase eta from undamaged human cells affects cell cycle progression and results in increased spontaneous chromosome breaks and CFS expression with the activation of ATM-mediated DNA damage checkpoint signaling [116].

Casper *et al*. [117] found that cells deficient in ATR, but not ATM, display a significant increase in CFS breakage following treatment with aphidicolin, compared to control cells. Importantly, CFSs are observed in ATR-deficient cells without aphidicolin induction. While the loss of *ATM* alone does not cause increased CFS expression, it is involved in maintaining CFS stability in the absence of ATR. Cells deficient in both ATR and ATM exhibit a significant increase in CFS breakage compared to those deficient in *ATR* alone [118]. Wan *et al*. [119] reported that ATR preferentially interacts with CFS FRA3B and the binding requires its kinase activity in response to aphidicolin treatment.

Some downstream targets of *ATR* are also involved in maintaining CFS stability. Loss of *CHK1*, but not *CHK2*, induces breaks at CFSs [120]. Inactivation of *HUS1*, a member of PCNA-related 9-1-1complex that promotes CHK1 phosphorylation by ATR and is involved in DNA repair, causes increased chromosomal instability at CFSs [121]. SMC1 is necessary for sister chromatid cohesion and DNA repair. Inhibition of SMC1 expression by RNAi is sufficient to induce fragile site expression [122]. Other proteins involved in checkpoint and DNA repair processes, such as BRCA1 [123], claspin [124], and FANCD2 [125], are required for CFS stability. Fanconi anemia (FA) is a recessively inherited syndrome with an extremely elevated cancer risk. Schoder *et al*. [126] showed that chromosomal break-points in Fanconi anemia patients co-localize on the molecular level with fragile sites in at least 50% of cases, supporting the involvement of FANC family in regulating CFS stability. In addition**,** CDC25A phosphatase, an essential component of the cell cycle machinery, is overexpressed in breast carcinoma. Constitutively overexpressed CDC25A in hTERT-immortalized primary human mammary epithelial cells causes defective DNA damage response and increased fragile site breakages [127].

The aphidicolin-induced DNA damage is repaired primarily by homologous recombination (HR), and *RAD51*, one of the key players in HR, participates in CFS stability. The *RAD51* silencing causes a broader distribution of breaks at CFS FRA16D than that observed with aphidicolin. Treatment with aphidicolin of *RAD51*-silenced cells further increases DNA breaks at CFSs. In contrast, the RNAi-mediated silencing of *PARP-1* does not cause chromosomal breaks or affect the expression/distribution of CFS induced by aphidicolin. Another DNA repair pathway, the mismatch repair (MMR), is also involved in CFS stability, mediated by *RAD51* [128]. Depletion of essential replication proteins in yeast leads to spontaneous DNA damage, genome rearrangements and breakpoints at fragile sites, which are subsequently repaired by HR [129].

Notably, proteins that are important for the resolution of DNA secondary structures, such as helicase and topoisomerase I, are capable of modulating CFS stability [130–134]. The Werner syndrome protein (WRN) is a member of the RecQ helicase family that is essential in maintaining CFS stability [130]. Murfuni *et al*. [133] showed that the role of WRN in response to perturbation of replication along CFS is functionally distinct from that carried out at stalled forks genome-wide. WRN modulates human DNA polymerase delta-dependent replication dynamics within the common fragile site FRA16D [134]. Topoisomerase I is a key enzyme functioning at the interface between DNA replication and mRNA transcription. Cells deficient in Topoisomerase-I accumulate stalled replication forks and chromosome breaks in S phase, and breaks occur preferentially at gene-rich regions of the genome. These defects could be mimicked by depletion of the splicing factor ASF/SF2 (alternative splicing factor/splicing factor 2), which interacts functionally with Topoisomerase I [131]. This study suggests that interference between replication and transcription causes spontaneous replication stress, leading to genomic instability during the early stages of tumorigenesis. However, there has been a debate about the role of topoisomerase-I in CFS expression. Arlt and Glover [132] reported that aphidicolin-induced breaks at CFSs are prevented when cells are co-treated with low concentrations of the topoisomerase I inhibitor, camptothecin. Furthermore, camptothecin reduces spontaneous fragile site breakage in ATR-deficient cells, even in the absence of aphidicolin. These results indicate that topoisomerase I activity is required for CFS breaks [132]. The contradictory conclusions about topoisomerase I and CFS instability imply the complexity of regulatory mechanisms underlying CFS expression. Whether camptothecin has other cellular targets, besides topoisomerase I, remains to be investigated. Although more investigation is necessary to better understand the mechanisms of CFS expression, it is clear that components of DNA damage checkpoints regulate CFS expression. Perturbed DNA synthesis and replication, in addition to interfered coupling of fork progression and transcription, causes DNA breaks preferentially occurring at CFSs.

## 5. An Active Role of CACGs in Maintaining Genomic Stability

A CACG, *FATS*, plays an active role in regulating checkpoint functions after DNA damage. Knockdown of *FATS* by siRNA causes more pronounced phosphorylation of Chk1, a mediator of DNA damage signaling [82]. In addition, the number of IR-induced nuclear 53BP1 foci in MEF cells is increased after *FATS* knockdown, indicating that FATS deficiency causes increased sensitivity to DNA damage induced by IR in normal cells. Furthermore, *FATS*-inhibited MEF cells exhibited significantly higher mitotic index after DNA damage [82]. For those FATS-deficient cells entering mitosis under IR-induced genotoxic stress, severe mitotic defects in nuclear division and centrosome duplication occur, confirming that *FATS* is required for sustaining G_2_/M checkpoint after DNA damage [82]. In agreement with the role of FATS as a tumor suppressor, the overexpression of FATS suppresses tumor growth both *in vivo* and *in vitro*. In an effort to determine the full length of *FATS* mRNA by screening the mouse testis cDNA library, the 5′-untranslated region (UTR) was validated, leading to the identification of FATS as a p53 target gene. Moreover, FATS is capable of suppressing tumor growth independently of p53 [107].

*N*-terminal domain of FATS (FATS-N), which consists of 363 amino-acid residues encoded by one exon that is highly susceptible to IR-induced deletion in tumors, is sufficient to induce p21 protein and inhibit cell proliferation. Interestingly, FATS does not affect the protein level of p27, another inhibitor of cell cycle progression, and FATS is capable of increasing p21 protein level in p53-null cells [82]. As p21 is an unstable protein and FATS does not possess features of a transcriptional factor, these results support a role of FATS in stabilizing p21. Indeed, the expression of FATS-N is sufficient to increase the half-life of endogenous p21 in both unstressed and stressed cells, in a p53-independent manner [82]. Li *et al.* further explored the mechanism by which FATS inhibited p21 turnover. Given that p21 is selectively induced by HDAC inhibitors and that HDAC1 is a major deacetylase, localized predominantly to the nucleus [135,136], a series of protein binding assays was performed to test whether FATS might interact with HDAC1. FATS (67–175) domain within FATS-N is required for protein interaction between FATS and HDAC1, which inhibits HDAC1 binding to p21 and facilitates the acetylation of p21. Li *et al.* first proved that acetylation of p21protein inhibits direct binding of p21 *C*-terminus to C8 α-subunit of 20S proteasome, suppressing subsequent ubiquitin-independent turnover of p21 [82]. In eukaryotic DNA damage signaling pathways, tumor suppressor p53 and its transcriptional target *CDKN1A* (p21) play an essential role in monitoring G_1_-S and G_2_-M cell-cycle checkpoints [137–139]. FATS therefore functions as a guardian to maintain genomic stability through, at least in part, regulating cellular abundance of p21 in response to DNA damage, independently of p53. More recently, there is evidence showing that FATS is a novel ubiquitin ligase that promotes p53 stabilization and activation in response to DNA damage [140] further supporting the role of FATS in regulating p53-p21 pathway to maintain genomic stability.

Ionizing radiation (IR) increases expression of *FATS* in cells carrying wild-type *p53*, which is in contrast to the response of *FHIT* and *WWOX* expression after DNA damage. Thavathiru *et al*. [141] reported that environmental carcinogens and ultraviolet (UV) light significantly downregulate expression of both *FHIT* and *WWOX* genes. Unexpectedly, IR does not affect expression of *FHIT* and *WWOX* genes. In contrast, aphidicolin-mediated replication stress induces tumor-like microdeletions in *FHIT*/FRA3B [142]. Whether *FHIT* and *WWOX* play a role in DNA damage response remains to be investigated.

## 6. Clinical Significance of CACGs

Several studies have investigated the clinical significance of CACG expression in cancer. Using an anti-FHIT polyclonal antibody in a standard immunohistochemical reaction, Lack of *FHIT* staining in a well-characterized cohort of 99 non-small-cell lung cancers (NSCLCs) was shown to be correlated with LOH at the *FHIT* 3p14.2 locus, and was inversely correlated with codon 12 mutations in K-ras. However, *FHIT* expression was not correlated overall with a variety of clinical parameters, including survival, and was not associated with abnormalities of immunohistochemical expression of p53 in the same cohort of NSCLC [143]. In an independent translational research on 58 primary and microdissected NSCLCs [144], *FHIT* LOH was not correlated overall with a variety of clinical parameters, including sex, smoking status, staging, lymph node metastasis and survival. There was no association between LOH at *FHIT* and its protein expression, suggesting the presence of complex mechanisms of *FHIT* inactivation. However, among 19 cases that showed LOH of *FHIT* detected by microsatellite marker D3S1766, a correlation between p53 overexpression and LOH at *FHIT* locus was observed [144], which differs from the previous conclusions [143]. The convincing conclusions about the correlation between FHIT and p53 expression may be obtained by quantitative real-time RT-PCR analysis in a large cohort. In the case of the *WWOX* gene, reduced WWOX expression demonstrates a significant association with clinical Stage IV (*p* = 0.007), negative Progesterone Receptor (PR) status (*p* = 0.008) and shorter overall survival (*p* = 0.03), by means of immunoblotting and immunohistochemistry on normal ovaries and specific human ovarian carcinoma tissue microarrays (*n* = 444) [145]. The expression level of *FATS*, determined by quantitative real-time RT-PCR, shows the clinical significance in breast cancer and NSCLC. In a cohort of 106 breast carcinomas, low expression of *FATS* is correlated with high nuclear grade. There is a tendency to a favorable outcome for patients with high expression of *FATS* (*p* = 0.346) in primary breast tumors. Interestingly, low expression of *FATS* was associated with a poor outcome of breast cancer patients with node positive (*p* = 0.011). Furthermore, the mRNA level of *FATS* showed an independent value in predicting the outcome of breast cancer patients with positive lymph nodes [108]. In addition, in a cohort of 89 NSCLC patients, a low level of *FATS* mRNA expression was correlated with poor overall survival in NSCLC (*p* = 0.030). For those NSCLC patients receiving cisplatin-based chemotherapy, the overall survival was significantly longer in the *FATS*-high subgroup than that in the *FATS*-low subgroup (*p* = 0.038). Multivariate analysis revealed the independent value of *FATS* mRNA in predicting the overall survival for NSCLC patients receiving cisplatin-based chemotherapy [109]. On the other hand, the clinical significance of oncogenic *CACG*s has also been shown. Met-regulated expression signature defines a subset of human hepatocellular carcinomas with poor prognosis and aggressive phenotype [146].

Although the potential value of CFS genes, such as cancer biomarkers, remains to be validated in a large cohort, CFS genes are emerging as a kind of biomarker with potential value in predicting response to chemotherapy or radiotherapy that cause DNA damage in both tumors and normal tissues. Given that CFSs are genomic “hotspots” of DNA damage, and DNA lesions at CFSs or defects in CFS gene expression are well correlated with chromosomal abnormalities in tumors, it would be interesting to evaluate whether monitoring deficiency of CFS-associated tumor suppressor genes may have a beneficial effect on optimizing the application of radiotherapy and radio-diagnosis, whose long-term side effects of carcinogenesis need to be weighed, particularly in the case of childhood [147–149].

## 7. Conclusions

CFSs are present in all individuals with varied frequency, representing late replicating AT-rich regions that are susceptible to DNA damage. In addition, CFSs are well conserved in mammals, and chromosome breakage and rearrangement at CFSs are early events in tumorigenesis. CFSs can be regarded as “built-in” sensors of DNA damage that link cell-cycle checkpoint to DNA repair pathways. However, only a few of CFS genes have been functionally validated. Future studies on CFS genes should enhance our understanding of tumorigenesis, leading to a more effective and personalized cancer therapy.

## Figures and Tables

**Table 1:** CACGs and molecularly mapped CFSs involved in cancer.

Human CFS	Location	Frequency	Associated genes	CACG
FRA2G	2q31	modest	*IGRP*, *RDHL*, *LRP2* and others	not validated
FRA2H	2q32	modest	non-coding RNA gene	not validated
FRA3B	3p14.2	high	*FHIT*	*FHIT*
FRA4F	4q22	modest	*GRID2*	not validated
FRA6E	6q26	modest	*PARK2*, *PLG*, *LPA* and others	*PARK2*
FRA6F	6q21	Modest	*REV3L*, *DIF13*, *FKHRL* and others	not validated
FRA7B	7p22	low	*THSD7A*, *SDK1*, *MAD1L1*	not validated
FRA7G	7q31.2	modest	*MET*, *TESTIN*, *CAV*, and others	*MET*, *TESTIN*
FRA7I	7q36	modest	*PIP*	not validated
FRA7K	7q31	modest	*IMMP2L*	not validated
FRA8C	8q24	modest	*MYC*	*MYC*
FRA9E	9q32	low	*PAPPA* and others	*PAPPA*
FRA10F	10q26	low	*FATS* and others	*FATS*
FRA10G	10q11	low	*RET*, *NCOA4*	*RET*
FRA16D	16q23.2	high	*WWOX*/*FOR*	*WWOX*
FRAXB	Xp22.3	modest	S*TS*, *GS1*	not validated
FRAXC	Xq22.1	modest	*DMD*, *IL1RAPL1*	*DMD*

**Table 2:** List of 13 BAC clones used for mapping of the proximal boarder of FRA10F.

BAC clone	Location on chromosome 10 (Mb)	Proximal to FRA10F	Supposed breakpoint	Inside FRA10F	Distal to FRA10F
RP11-198M6	121.577.828-121.765.053	1	-	-	-
RP11-323P17	122.469.430-122.626.379	1	-	-	-
RP11-105F10	123.764.471-123.939.147	1	-	-	-
RP11-296H2	123.939.148-124.083.531	1	-	-	-
RP11-436O19	124.267.798-124.268.644	1	-	1	-
RP11-481L19	124.268.654-124.444.805	1	-	-	-
RP11-564D11	124.592.597-124.787.891	1	-	-	-
RP11-162A23	124.799.045-124.978.916	1	-	-	-
RP11-391M7	125.381.493-125.576.300	1	-	-	-
RP11-435D11	125.909.463-126.009.423	-	1	-	-
RP13-238F13	126.009.424-126.190.394	-	-	1	-
RP11-124H7	127.445.890-127.599.588	-	6	-	-
RP11-179O22	128.076.478-128.246.854	-	1	3	-

Notes: BAC clones from 10q26.13–26.2 spanning 8 Mb were selected from the UCSC database (http://genome.ucsc.edu). The frequency of FRA10F in one individual was 4 in 500 metaphase spreads (0.8%) compared to 1 in 500 (0.2%) in the second individual. *FATS*-containing BAC clone RP11-179O22 was hybridized on four different metaphase spreads in FISH analysis, showing that *FATS* was located once at the breakpoint and three times within FRA10F. Therefore, the location of FRA10F is refined to 10q26.13–q26.2 (Figure 2).
